# Mental health consequences of long-term stays in refugee camps: preliminary evidence from Moria

**DOI:** 10.1186/s12889-021-11301-x

**Published:** 2021-07-02

**Authors:** Willemine van de Wiel, Carla Castillo-Laborde, I. Francisco Urzúa, Michelle Fish, Willem F. Scholte

**Affiliations:** 1 Moria Medical Support (MMS), Windroosplein 68b, 1018 ZW Amsterdam, The Netherlands; 2 Centro de Epidemiología y Políticas de Salud, Facultad de Medicina Clínica Alemana, Universidad del Desarrollo, Av. Las Condes 12, 438 Lo Barnechea, Chile; 3 Faculty of Finance, Bayes Business School, City University of London, 106 Bunhill Row, London, EC1Y8TZ UK; 4 Amsterdam UMC, Department of Psychiatry, University of Amsterdam, Meibergdreef 5, 1105 AZ Amsterdam, The Netherlands

**Keywords:** Refugees, Camp, Greece, Containment, Mental health, Crisis

## Abstract

**Background:**

Ever since the implementation of the EU-Turkey deal, most refugees that enter Greece via sea are confined to the island on which they arrive until their asylum claims are adjudicated, where they generally reside in camps. Some of these camps have detention-like characteristics and dire living conditions, such as Moria camp on the island of Lesbos, Greece. Aid-organizations have stated that the situation in camp Moria deteriorates the mental health of its inhabitants and there is qualitative evidence to support this. This study explores the quantitative relationship between the incidence of acute mental health crises and the length of stay in the camp.

**Methods:**

A cross-sectional study was conducted using routinely collected data on 856 consultations of 634 different patients during 90 nights at an emergency clinic in Moria camp. Logistic regression analysis was used to explore whether the length of stay in the camp was predictive of the occurrence of an acute mental health crisis.

**Results:**

Of the 634 patients, the majority were men (59·3%), the average age was 23·2 years [0–71], and 24·3% was < 18 years. 25·5% (*n* = 218) of consultations were related to mental health problems; 17·0% (*n* = 37) of these met the study’s case definition of an acute mental health crisis. Such crises were positively associated with the length of stay in the camp (*p* = 0·011); the odds ratio of a mental health crisis increases with 1·03 for every 10% increase in days of residence in the camp. This is notable when considering the average length of stay in the camp is 71 days.

**Conclusion:**

This study offers quantitative support for the notion that the adverse conditions in Moria camp deteriorate the mental health of its inhabitants as suggested in qualitative research. Although this study does not provide evidence of causality, it is likely that the poor and unsafe living conditions, challenging refugee determination procedures, and a lack of mental health services in the camp are significant contributing factors. We urgently call for Europe’s policymakers to honour the ‘51 Geneva refugee convention and terminate the neglectful situation on the Greek archipelago.

## Background

Over the last decade, millions of refugees have arrived in Europe, often by crossing the Mediterranean by boat or by traveling by land via Turkey, through Greece and the Balkans to Western Europe [1]. The number of people arriving rose slowly between 2010 and 2013 and then tripled in the 2 years that followed. In 2015, a record 1·3 million asylum applications were received in the EU [2], a situation that became known as the ‘migrant crisis’. In efforts to reduce immigration through the EU border-states, EU member states implemented various measures, notably the closure of the Balkan routes and the implementation of the EU-Turkey agreement in March 2016 [3].

While immigration via Greece substantially decreased following the EU-Turkey agreement, the length of stay for a refugee at the Greek entry locations drastically increased due to a prolonged administrative process [1]. Upon arrival, refugees generally reside in camps, which, even though originally designed as short-stay provision, became long-stay facilities. Some of these camps have detention-like characteristics and dire living conditions [4].

In accordance with the EU-Turkey deal containment policy, refugees are confined to the island on which they arrive until their asylum claims are adjudicated. Those who are deemed vulnerable (e.g. the elderly, sick, pregnant or those displaying severe mental health conditions) should be exempt from this policy and should be allowed to await the outcome of their asylum procedure on mainland Greece, where facilities are better [5]. However, the lack of accommodation on the mainland and delays in the vulnerability assessment procedure leave thousands of eligible individuals and families trapped on the island [5].

The island of Lesbos (or Lesvos) was, and still is a key entry point in Greece for refugees [6]. Hence, Lesbos houses several refugee camps, one of them being Moria camp, which has been repeatedly reported for being overcrowded, unhygienic, and unsafe [7–9] despite EU funding and the efforts of privately-funded non-governmental organizations [10, 11]. Charlie Yaxley, a spokesman for the UN High Commissioner for Refugees (UNHCR), reported the following about the camp in August 2018: “We are particularly concerned about woefully inadequate sanitary facilities, fighting amongst frustrated communities, rising levels of sexual harassment and assaults, and the increasing need for medical and psychosocial care” [12].

In Moria camp, few refugees feel safe and well-treated [13]. Psychological problems are omnipresent, and rates of attempted suicide are high [14]. Health care access is poor, especially for mental health care, and services are mostly provided by a changing group of medical volunteers [15, 16]. Multiple aid organizations have raised concerns about what they describe as a mental health crisis [7, 17]. They have pressed the EU member states and the Greek government to improve living conditions and decongest the island, arguing that the camp conditions negatively affect the mental health of its inhabitants [18].

The claim that the camp conditions like those in Moria adversely affect mental health has some empirical support. It has been well established that, compared to the general population, the prevalence of mental health conditions (in particular PTSD, anxiety, and depression) is higher in refugees and other conflict-affected populations [19] due to pre-migration stressors [20]. However, a growing number of studies in recipient countries found that imposed conditions of adversity, including prolonged detention or living in institutional accommodation, uncertain residency status, challenging refugee determination procedures, restricted access to services, and a lack of opportunities to work or study, combined in a way that compounded the effects of past traumas in exacerbating symptoms of PTSD and depression in this population [21, 22]. In a series of studies, Miller et al. stressed the mediating role of daily stressors such as unsafe living conditions, challenges in meeting basic survival needs (access to water, food, shelter, healthcare), the inability to produce income, and isolation from family and traditional social supports, on the effect of past war-related trauma on mental health in refugees [23–25]. In a study in two Rohingya refugee camps, it was found that, while PTSD symptoms were associated with both prior trauma exposure and environmental stressors *(*problems with food, lack of freedom of movement, and concerns regarding safety), depression symptoms were associated with daily stressors only [26]. Studies that focus on the relation between the length of time spent in the asylum procedure (be it in immigration detention, a refugee camp, or another institutional accommodation) and mental health find a cumulative adverse effect [27–29]. In turn, release from detention or being granted a permanent visa improves mental health [30, 31].

Two qualitative studies examining the link between Moria camp conditions and mental wellbeing both suggest that camp conditions (lack of safety and proper living conditions, institutional abuse, slow and constantly changing asylum procedure, lack of (mental) health service provision, lack of functional and supportive networks) lead to a deterioration in mental health [32, 33]. This effect, however, has never been quantified.

This study aims to establish the relationship between the length of stay in Moria camp and mental health by using quantitative data provided by Moria Medical Support (MMS), a Dutch-registered medical NGO. MMS provided emergency medical care in Moria between nine o’clock in the evening and eight o’clock in the morning, 7 days a week. As expected, working conditions were challenging because of limited resources and a lack of safety. See Table 1. We hypothesized that given the prevailing stressors (lack of safety, challenges in access to water, food, shelter, and healthcare, inability to produce income, lack of supportive networks, institutional abuse, and uncertainty regarding the length of the asylum procedure), a longer length of stay in the camp would negatively impact mental health.

**Table 1:** The clinic's working conditions

The clinic consisted of a container with three small consultation rooms. Unable to gain medical access during the day, patients of all ages and nationalities would line up well before opening hours. A triage system was used to ensure that the worst cases were addressed, adding to desperation and anxiety in those turned away. Inside the clinic, there was often agitation and noise; earplugs were sometimes handed to patients to prevent panic or dissociation from being triggered. MMS staff had to lock themselves in and call the police on a regular basis, mostly because of threatening or aggressive patients. On one occasion, 20 armed men attacked the clinic, trying to assault a patient who was inside. The night watch of the police was outnumbered and could not prevent the clinic from being demolished and patients and staff inside being assaulted. It marked the end of the clinic’s activities.	

Restricted by the nature of clinical activities at the MMS clinic and the healthcare situation in the camp, we focused on the relationship between length of stay and the incidence of acute mental health crises rather than mental health conditions in general. This will further be clarified in the Methods section.

Ethical approval was waived by the Ethics Committee of Santiago’s Universidad del Desarrollo, Chile, as the study only made use of data from secondary sources, routinely collected, anonymized information on patients.

## Methods

### Study site and period

The night clinic of MMS provided emergency medical care for all residents of Moria from January to April 2018 (when the clinic was demolished), 21:00 to 08:00 h, 7 days per week. Data used for this study were collected over this period. During this period, the camp population size fluctuated between 5560 and 6429 people [34]. Children (< 18 years) constituted at least 30% of the camp population [35].

### Dataset

An anonymized database was provided by MMS, comprising the routinely collected information on 1206 clinical consultations with 937 unique visitors on 90 different days. For some patients, relevant data were missing, meaning that the final sample comprised 856 consultations from 634 unique patients. Apart from medical details (problem, diagnosis, and treatment), information was documented on patients’ gender, age, country of origin, and an indication of the length of stay in the camp at the time of consultation. Categorization of raw medical data was done in retrospect by the first author of this article.^1^

### Study design and data analysis

In this cross-sectional study, in addition to descriptive analysis, logistic regression analysis was performed using the length of time in the camp in days as an input variable, together with age (in years), gender (dichotomous variable, one if male), and country of origin as possible confounders. The output variable was ‘acute mental health crisis’. As substantiated in the below paragraph ‘Dependent variable’, ‘acute mental health crisis’ was defined for this study as encompassing three conditions: a) self-harm, as constituted by a non-accidental self-inflicted wound, the majority being skin cutting; b) a suicide attempt as constituted by an action with suicidal intent, in which either the seriousness of the intent or the resulting injuries warranted immediate referral to (in-hospital) specialized care; c) a severe state of panic and/or agitation and/or dissociation and/or psychosis as constituted by anxiety, nervous agitation, undirected aggression, alienation or disturbed reality testing, causing disturbed behavior resistant to non-invasive treatment (i.e., adequate psychological approach, relaxation and grounding techniques, or/and oral psychotropic medication) and thus requiring intramuscular psychotropic medication.

Demographic (age and gender) and displacement characteristics (country of origin) were described, as well as a general overview of the mental health consultations. The analysis considered the average, minimum, and maximum for continuous variables (age and length of stay) and the percentage distribution for categorical variables (country of origin and causes of consultation).

Two sets of logistic regressions were conducted with all the consultations for which we have complete data using the manifestation of any of the conditions covered by this study’s case definition as a dependent variable (value of one if present, zero otherwise). Trying to understand the importance of each of our demographic characteristics, in our first set of regressions, we conducted a bivariate analysis, including each of these variables independently. We then regressed our displacement characteristics, a set of dummies for the country of origin. In our second set, the multivariate analysis, we focus on the duration of stay as an independent variable, while also including country of origin, age and gender, as the prevalence of mental health disorders vary with gender [36] and through individuals’ lifespan [37, 38]. All statistical analyses were performed using STATA version 16 software.^2^

### Dependent variable

A mental health crisis can be defined as any situation in which a person’s behavior puts them at risk of hurting themselves or others, or prevents them from being able to care for themselves and function effectively in the community [39]. It can be seen as the manifestation of an exacerbated mental health condition or of the sudden collapse of an unstable psychological balance. As a case definition, we used the three conditions mentioned in the first part of the ‘Study design and data analysis’ paragraph above.

The focus on the relationship between the length of stay and the incidence of acute mental health crises rather than on mental health conditions in general has two main reasons. First: the number of mental health conditions identified at the MMS clinic could not be expected to be indicative of the incidence of mental health conditions in the camp population for various reasons. Help-seeking behavior depended on many factors such as demographic variables like gender and age [40], on cultural background [41], and on safety as experienced during the nightly opening hours of the clinic. The clinic was known to offer only Psychological First Aid (PFA) and psychiatric crisis management. Patients who were not in crisis were sensitized to visit psychosocial programs by other NGOs during the daytime. Only rarely short-term access to mental health services (like that of Médecins Sans Frontières, MSF) was negotiated since only the worst cases were accepted. As a result, non-urgent patients might not have been inclined to revisit the clinic unless they were (close to) being in crisis. A second reason to focus on mental health crises as the dependent variable was the fact that few of the volunteer medical staff at the MMS clinic were qualified in psychiatric diagnosing and even if they were, there was simply no time for a comprehensive assessment.

In addition, using acute mental health crises as the dependent variable comes with advantages. The association between the presenting rate of acute mental health crises at the clinic and the incidence of such crises in the camp is likely to be strong, as patients with serious suicide attempts, significant self-harm, and severe panic/agitation/dissociation/psychosis tended to be brought to the clinic by bystanders if they would not come on their own accord. We added ‘resistant to non-invasive treatment’ to the third condition to differentiate between relatively mild or self-limiting crises (patients that might have stayed at their dwelling) and more serious cases (patients that were likely to be brought to the clinic by the community if not coming on their own accord). Moreover, the identification of an acute mental health crisis in the dataset is straightforward, despite the diagnostic limitations of the clinic; symptoms were overt, treatment was invasive (injection/stitching), or presentations warranted immediate referral.

## Results

Table 2 shows the demographic and displacement characteristics for the refugees in the study sample (*n* = 634). The majority were men (59·3%) of Syrian (31·4%) or Afghan (26·5%) origin, while the average age was 23·2 years [0–71], and 24·3% were children (< 18 years). The average length of stay in the camp until consultation was 70·9 days [0–532]. A total of 143 individuals presented with mental health problems. Those with an acute mental health crisis (*n* = 25) were mostly Iraqi (42·3%) men (88·0%), with an average age of 23·8 years [15–36]. The average length of stay until consultation for this group was 105·9 days [2–471]. Individuals presenting with non-acute mental health problems (*n* = 118) were mostly from Democratic Republic of Congo - DRC (52·5%), male (69·5%), with an average age of 24·9 [12–66], and an average length of stay until consultation of 67·9 days [1–532].

**Table 2:** Refugee demographic and displacement characteristics

Refugee characteristics	Total (*n* = 634)	Acute mental health crisis (*n* = 25)	Other mental health presentations (*n* = 118)
Nationality (%)			
Syria	31.4%	15.4%	16.1%
Afghanistan	26.5%	11.5%	12.7%
Iraq	20.7%	42.3%	16.9%
Democratic Republic of Congo	15.8%	7.7%	52.5%
Iran	3.8%	7.7%	0.8%
Other	1.9%	15.4%	0.8%
Male	59.3%	88.0%	69.5%
Age (years)	23.2 [0–71]	23.8 [15–36]	24.9 [12–66]
Children (< 18 years) (%)	24.3%	4.0%	7.6%
Stay until consultation (days)	70.9 [0–532]	105.9 [2–471]	67.9 [1–532]

Some individuals presented more than once with mental health problems, whether or not acute. The individuals with mental health problems (*N* = 143) had in total 218 mental health-related consultations (25·5% of all consultations) (Table 3). Acute mental health crises represented 17·0% (*n* = 37), with self-harm (6·9%) and severe panic/agitation/dissociation/psychosis (6·4%) being the most common, followed by suicide attempt (3·7%). The most relevant other mental health consultations (83·0%) were trauma-related symptoms (28·9%) and mild to moderate panic/agitation/dissociation/psychosis (23·4%). There was no significant difference between included and excluded consultations in terms of incidence of acute mental health crises.

**Table 3:** Type and frequency of mental health consultations

Type	Total (*n* = 218)
Acute mental health crisis	17.0%
Severe Panic/Agitation/Dissociation/Psychosis	6.4%
Self-harm	6.9%
Suicide attempt	3.7%
Other mental health presentations	83.0%
Trauma related symptoms	28.9%
Mild to moderate Panic/Agitation/Dissociation/Psychosis	23.4%
Low mood and/or suicidal ideation	11.9%
Other	18.8%

Table 4 presents the results of a logistic regression analysis we performed with each of our demographic and displacement characteristics independently, showing that the male gender is significantly associated with increased odds of acute mental health crises, whereas age does not seem to increase these significantly. Length of stay (days in log) increases the odds of acute mental health crises, with a 10% increase in the number of days increasing the odds ratio by 1·032. The last column shows that refugees from DRC are less likely to suffer acute mental health crises, with the opposite being true for those from Iran, Iraq and Syria.

**Table 4:** Logistic regression odds ratios (OR), 95% confidence intervals [in brackets] and *p*-values for various refugee characteristics (left column) as predictors of acute mental health crisis (right columns)

Refugee characteristics	Acute mental health crisis odds ratio, [95% confidence limits], *p* value			
Male	5∙945			
	[1∙159–30∙489]			
	0∙033			
Age		1∙002		
		[0∙991–1∙013]		
		0∙723		
Length of stay until consultation (days in log)			1∙393	
			[1∙118–1∙737]	
			0∙003	
Country of origin Democratic Republic of Congo				0∙872
				[0∙872–0∙872]
				0∙000
Country of origin Iran				4∙572
				[4∙572–4∙572]
				0∙000
Country of origin Iraq				4∙420
				[4∙420–4∙420]
				0∙000
Country of origin Syria				1∙289
				[1∙289–1∙289]
				0∙000
Other countries of origin				Yes

In Table 5, the multivariate analysis includes all of our demographic and displacement characteristics. The results are consistent with the univariate analysis. The length of stay in Moria camp (in log) is significantly associated with increased odds of 1·402 ([1∙081–1∙819]; *p* = 0·011. A 10% increase in the number of days spent at the camp increases the odds ratio by 1·033. There are still decreased odds with DRC as a country of origin and increased odds with Iran, Iraq, and Syria as the country of origin. The factor of gender lost its significance, and age is still not significantly associated with the odds of an acute mental health crisis.

**Table 5:** Logistic regression odds ratios (OR), 95% confidence intervals [in brackets] and *p*-values for various refugee characteristics (left column) as predictors of acute mental health crisis (right columns)

Refugee characteristics	Acute mental health crisis odds ratio, [95% confidence limits], *p* value		
Male	4∙607	[0∙739–28∙722]	0∙102
Age	1∙002	[0∙992–1∙013]	0∙675
Length of stay until consultation (days in log)	1∙402	[1∙081–1∙819]	0∙011
Country of origin Democratic Republic of Congo	0∙726	[0∙601–0∙877]	0∙001
Country of origin Iran	4∙307	[3∙402–5∙453]	0∙000
Country of origin Iraq	3∙937	[3∙294–4∙707]	0∙000
Country of origin Syria	1∙238	[1∙093–1∙402]	0∙001
Other countries of origin		Yes	

## Discussion

This cross-sectional study among refugees in Moria camp on Lesbos, Greece, examined the incidence of acute mental health crises and their relationship with the length of stay in the camp. The data used were collected from 856 consultations during 90 nights at an emergency night clinic in the camp. While 25·4% (*n* = 218) of the consultations were related to mental health problems, 17·0% (*n* = 37) of these met the study’s case definition of acute mental health crisis.

Acute mental health crises were significantly and independently (the multivariate analysis corrects for interaction between the input variables) associated with the length of stay in the camp (*p* = 0·011). The established association implies that the odds ratio of experiencing such crisis increases with 1·033 for every 10% increase in days of residence. This is significant when considering the average length of stay in the camp (70·9 days). Other factors associated with acute mental health crises were Iranian/Iraqi/Syrian ethnicity, augmenting the risk, while Congolese ethnicity reduced the risk. The male gender was significantly associated with a crisis in the univariate analyses, but gender lost its significance in the multivariate model (*P* = 0·102). We failed to find a significant association between acute mental health crises and age 38.

The limited information in the database prevented us from investigating prior mental illness as a possible confounder. However, we deem it very unlikely that the found correlation between acute mental health crisis and the length of time spent in the camp is positively impacted by prior history. If anything, it would reduce the size of this correlation. Patients with a severe physical or mental condition are granted a vulnerability status that entitles them to await the outcome of their asylum application in another facility. Although the transfer is not always effectuated, it is safe to assume that a prior mental health condition does not prolong the duration of the time spent in the camp. Furthermore, one would expect refugees with a previous history of mental illness to suffer acute crises more often than those without, meaning that they would likely have presented themselves after a shorter length of stay in the camp.

This study offers quantitative support for the notion that Moria camp deteriorates mental health, as suggested by qualitative reports in scientific journals [32, 33], and communications by aid workers [7, 18]. Our findings are in line with previous research investigating the effect of post-migration stressors in refugee facilities on mental health, as discussed in the introduction. Furthermore, by focusing on acute mental health crises, our study extends the existing evidence related to the prolonged asylum process’s detrimental mental health effects due to adverse living conditions. Exacerbations of mental health conditions do not only add to the suffering of the affected individuals, but also to ‘collateral damage’ in various forms, ranging from serious destabilization of the social environment to physical harm to others in the person’s environment, all of which may traumatize an already vulnerable population, children in particular [17]. While the current policy detains refugees at Lesbos, the negative mental health implications of immigration detention [28] are believed to continue after release [42], eventually affecting successful integration in future host societies [43].

Our study also provides some understanding of the magnitude of the problem. Thirty-seven acute mental health crises in 3 months on a population of circa 6000 inhabitants may not seem remarkable; this number, however, reflects only a minor part (17%) of the total mental health presentations that were selected through the triage system of the overnight emergency clinic. When one would extrapolate this presentation rate to the context of a small European hospital (catering for a population of 100,000 inhabitants), it would compare to 40 mental health presentations per night, amongst them 3 cases of self-harm, 2–3 patients needing invasive drug treatment and 1–2 serious suicide attempts.

These substantial numbers are likely to be more than an effect of premigration trauma and the asylum procedure, but also a result of the poor and unsafe living conditions, challenging refugee determination procedures, and a lack of mental health services in the camp, as explained in the Introduction section of this article. It also indicates that access to mental health care during the day fell seriously short, as was already suggested by qualitative reports [15, 16, 32].

The observation that the risk of an acute mental health crisis varies amongst refugees of different nationalities cannot be directly linked to past literature. Elaborating on possible causes falls well beyond the scope of this article but might be interesting for follow up research. That the male gender seems to predispose to an acute mental health crisis (although not significantly so in this study), complies with literature. Although women (refugees and non-refugees alike) are reported to suffer more from anxiety and depression and perform more deliberate self-harm [44–46], serious suicide attempts, completed suicides and admissions to a psychiatric intensive care unit are more common in men [47–49]. In addition, men in general show higher levels of externalizing (disruptive and violent behavior) and substance abuse [50] and refugee men have an increased risk of psychosis [51]. Although expected, a relation between age and acute mental health crisis could not be objectified in our model. This may be the effect of the non-linear relation of age with mental health morbidity [37, 38].

This study, based on real-world frontline data, gives an impression of the level of acute distress experienced by refugees stranded on the Greek islands. However, it has several limitations, mostly related to data availability. An initial limitation is that the average length of stay for the camp inhabitants could not be quantified, as no data could be collected on a population level, which might have led to an unavoidable selection bias. A second limitation is that there was probably a higher incidence of acute mental health crises than the one estimated in this study. As described in Table 1, medical documentation might have been problematic due to difficult working conditions, for example patients being too aggressive and/or intoxicated, meaning that not all refugees suffering an acute mental health crisis were included. Furthermore, acute mental health crises that were professionally addressed during the daytime clinic hours were not seen by MMS, thus not included in the study sample. Lastly, no data exists on actual suicides. Given the serious nature of some attempts reported or identified at the night clinic, one might assume other attempts to have resulted in death. Relevant data, however, were not available.

Another limitation was methodological in nature. The concept of an ‘acute mental health crisis’ is usually not defined in terms of pathology [52, 53], but by the involvement of an ambulant crisis team or hospital admission of a patient. Due to the near absence of mental health services for the camp inhabitants, the diagnostic limitations of the clinic’s personnel, and the summary information in the database, such a definition would not suffice. With the pragmatic but narrow definition operated for this study, however, some patients were excluded that would under normal circumstances be admitted to a mental health facility.

We believe that a camp-wide cross-sectional or prospective cohort study design with standard psychiatric interviewing would have been preferable in terms of methodology. However, it was not possible to conduct such a study for the authors of this article. The main reasons were our limited means (no funding) and capacity (limited qualified personnel), the organization of the camp, and the political sensitivity. There was no funding to perform a camp-wide survey followed by the necessary psychological follow up in this vulnerable population. Furthermore, while conducting a survey would require permission from the Greek army, which formally directed Moria camp, Greek authorities have not been forthcoming with exposing the camp conditions. Only a handful of NGOs were allowed access to the camp premises, media were generally banned, and journalists have even been arrested on instigation of the Ministry of Defense after publishing information related to Moria camp [54].

To date, facilities for refugees on Lesbos and the other Greek islands have not improved since the EU-Turkey deal, which was signed almost 5 years ago. Meanwhile, camp populations have tripled or quadrupled, depleting not only the stamina of the refugees, but also exasperating island inhabitants. We hope this paper adds to the understanding of the adverse consequences of the current situation for the medical world and Europe’s policymakers.

## Conclusion

This study, based on frontline medical data collected in 2018, offers quantitative support for the notion that the adverse conditions in Moria camp (Lesbos, Greece) lead to a deterioration in mental health, as suggested by qualitative studies and in communications by aid workers. It also offers an insight into the magnitude of the mental health burden in the camp as well as the lack of mental health services.

While acknowledging that this study does not provide evidence of a causal relationship between the length of stay and mental health crises, it is fair to suggest that the poor and unsafe living conditions, challenging refugee determination procedures, and a lack of mental health services in the camp are significant contributing factors. We urgently call for Europe’s policymakers to honor the ‘51 Geneva refugee convention and terminate the neglectful situation on the Greek archipelago.

### Post scriptum

On the ninth of September 2020, during this manuscript’s revision process, Moria camp was largely destroyed by a fire believed to have been set by some of its inhabitants. The perpetrator(s) might be portrayed as ungrateful and dangerous refugees, trying to manipulate their way out of the island. We hope this paper sheds a different light on the matter. Although fire-setting in a refugee facility is not uncommon [55, 56], it is usually performed by outsiders. As a matter of self-interest, people do not set fire to their place of refuge, putting their own and other lives in danger. However, at Lesbos, refugees found despair next to refuge. Here, the criminal act of arson may have acted as the expression of utter despair.

## Data Availability

Datasets analyzed for the current study were provided by MMS. Requests for data sharing can be directed to the corresponding author, who will discuss the meeting of requests with MMS board members.

## Abbreviations

MMS: Moria Medical Support (a Dutch registered Medical NGO)
EU: European Union
UN: United Nations
UNHCR: United Nations High Commissioner for Refugees
NGO: Non-governmental organization
PTSD: Post-traumatic stress disorder

## Footnotes

1 As a robustness test, the categorization for acute mental health crisis was double-checked by WFS and we re-ran our main analysis, with results (odds ratio’s) remaining unchanged.

2 We performed two robustness tests (King and Zeng’s rare event logit and Poisson regression), as it could be argued that the event’s frequency is relatively low. Our results remained unchanged.
